# The state of emergency obstetric care services in Nairobi informal settlements and environs: Results from a maternity health facility survey

**DOI:** 10.1186/1472-6963-9-46

**Published:** 2009-03-12

**Authors:** Abdhalah K Ziraba, Samuel Mills, Nyovani Madise, Teresa Saliku, Jean-Christophe Fotso

**Affiliations:** 1 African Population and Health Research Center, P.O. Box 10787, 00100, Nairobi Kenya; 2 World Bank, The World Bank, MSN G7-701, 1818 H Street NW, Washington, DC 20433, USA; 3 University of Southampton, School of Social Sciences, University of Southampton, Southampton, SO17 1BJ, UK; 4 Faculty of Health & Applied Social Sciences, Center for Public Health, Liverpool John Moores University, 8 Marybone, Liverpool, L3 2AP, UK

## Abstract

**Background:**

Maternal mortality in Sub-Saharan Africa remains a challenge with estimates exceeding 1,000 maternal deaths per 100,000 live births in some countries. Successful prevention of maternal deaths hinges on adequate and quality emergency obstetric care. In addition to skilled personnel, there is need for a supportive environment in terms of essential drugs and supplies, equipment, and a referral system. Many household surveys report a reasonably high proportion of women delivering in health facilities. However, the quality and adequacy of facilities and personnel are often not assessed. The three delay model; 1) delay in making the decision to seek care; 2) delay in reaching an appropriate obstetric facility; and 3) delay in receiving appropriate care once at the facility guided this project. This paper examines aspects of the third delay by assessing quality of emergency obstetric care in terms of staffing, skills equipment and supplies.

**Methods:**

We used data from a survey of 25 maternity health facilities within or near two slums in Nairobi that were mentioned by women in a household survey as places that they delivered. Ethical clearance was obtained from the Kenya Medical Research Institute. Permission was also sought from the Ministry of Health and the Medical Officer of Health. Data collection included interviews with the staff in-charge of maternity wards using structured questionnaires. We collected information on staffing levels, obstetric procedures performed, availability of equipment and supplies, referral system and health management information system.

**Results:**

Out of the 25 health facilities, only two met the criteria for comprehensive emergency obstetric care (both located outside the two slums) while the others provided less than basic emergency obstetric care. Lack of obstetric skills, equipment, and supplies hamper many facilities from providing lifesaving emergency obstetric procedures. Accurate estimation of burden of morbidity and mortality was a challenge due to poor and incomplete medical records.

**Conclusion:**

The quality of emergency obstetric care services in Nairobi slums is poor and needs improvement. Specific areas that require attention include supervision, regulation of maternity facilities; and ensuring that basic equipment, supplies, and trained personnel are available in order to handle obstetric complications in both public and private facilities.

## Background

High levels of maternal mortality in sub-Saharan Africa remains a challenge. The WHO/UNICEF/UNFPA estimated maternal mortality ratio in Kenya at 1,000 maternal deaths per 100,000 live births in 2000[1] Of the two indicators (maternal mortality ratio and proportion of births with skilled attendants) for measuring progress towards the fifth Millennium Development Goal (MDG5) of reducing maternal mortality between 1990 and 2015 by three quarters[2], maternal mortality ratio is generally more difficult to measure compared to the proportion of births with skilled birth attendants (doctors, midwives, or nurses) which can readily be measured in national surveys[3,4]. Knowing the proportion of women who deliver with skilled assistance is not enough. Mere presence of skilled attendants at birth is unlikely to reduce maternal mortality if there is no supportive environment with essential drugs and supplies, equipment, and appropriate referral and communication system [5].

Increasingly it is being recognized that availability and access to emergency obstetric care improves maternal morbidity and mortality[6,7]. Based on functionality and ability to provide lifesaving emergency obstetric procedures, a health facility can be classified as either basic or comprehensive emergency obstetric care facility (EmOC) [8] Basic EmOC facilities are expected to provide the following six services: administration of parenteral antibiotics; parenteral oxytocic drugs; parenteral anticonvulsants for pre-eclampsia; manual removal of retained placenta; removal of retained products of conception; and assisted vaginal delivery (vacuum extraction or forceps delivery). Comprehensive EmOC facilities are expected to provide caesarean section and blood transfusion in addition to those services provided by the basic EmOC facilities.

In 2004, the Kenya Service Provision Assessment (KSPA) survey among other things examined the availability of emergency obstetric care and quality of delivery services [9]. The KSPA categorized EmOC facilities in Kenya as basic EmOC "minus 1" (excluding assisted vaginal delivery) and comprehensive EmOC "minus 1" (excluding assisted vaginal delivery). Out of 1,882 health facilities (hospitals, maternity clinics and health centers) in Kenya at the time of the survey, it was estimated that 9% offered basic EmOC "minus 1" while 6% offered comprehensive EmOC "minus 1". There was geographical variation among the 8 provinces with Nairobi province having the lowest number of comprehensive EmOC "minus 1" per 500,000 population (0.4) while the Coast province had the highest at 3.8 comprehensive EmOC "minus 1" per 500,000 population. The low coverage in Nairobi province might be partly due to the ever growing population in the city's slums without concomitant expansion of health services. It is estimated that over 70% of the population of Nairobi city live in slums [10]. The KSPA did not provide a break down on the status of EmOC in Nairobi slums.

Recent studies show that the urban poor are increasingly becoming disadvantaged in terms of health outcomes. A study conducted by the African Population and Health Research Center (APHRC) in 2000 showed that most health indicators in the Nairobi slums were worse than other parts of Kenya including rural areas. Under-five mortality in Nairobi's slums was 151 deaths per 1000 live births compared to 84 deaths per 1000 live births in other urban areas of Kenya, and 113 in rural areas. Forty-four percent of children received full vaccination in Nairobi slums compared to 69% in other urban areas and 64% in rural Kenya [11]. Typical characteristics of Nairobi slums include high unemployment; poor access to social amenities such as housing, water, education; and inadequate health provision[10,12]. Rapid growth of the urban poor population, which has surpassed growth in social services including health facilities, poses a challenge to planners. Whereas physical distance might be the biggest hindrance to accessing health care in rural areas, other factors including cost and congestion in government facilities are often major barriers to the utilization of services in urban areas. Thus, if progress in health and development is to be achieved in Africa, the global community needs to pay attention to the growing urban poor population. This paper assesses quality of emergency obstetric services available to women in two typical Nairobi slums, Korogocho and Viwandani with specific reference to staffing levels and skills, equipment, drugs and supplies, information management, and referral facilities.

## Methods

The data used in this paper came from a maternal health research project conducted by APHRC in 2006. APHRC is a non-governmental research institution with a focus on health and population challenges facing the African continent. APHRC maintains a longitudinal surveillance system referred to as the Nairobi Urban Health and Demographic Surveillance System (NUHDSS) in two slums in Nairobi city where a total of about 60,000 individuals living in 23,000 households are under surveillance. Vital events monitored include births, deaths and movements among others. The NUHDSS also provides a platform for most of the Center's research projects.

The health facility survey from which this paper is derived was one of the five components of the bigger maternal health project. The other four were: a household survey of women who had pregnancy outcomes between 2004 and 2005; in-depth interviews with women who experienced severe complications (near misses) during their last pregnancy; focus group discussions of women and men regarding delivery experiences; and verbal autopsy interviews for female deaths for the period of 2003–2005. Only details of the health facility survey are presented in this paper. The criteria for inclusion of health facilities in the survey were: provision of delivery care to the slum population in Korogocho and Viwandani as reported by the women who participated in the household survey component of the project, and location or proximity to the study site. Out of the 37 health facilities that were mentioned by women as delivery places, interviews were successfully conducted in 25 health facilities. Two facilities declined to participate while ten health facilities mentioned by women as providing delivery services reported not to be providing maternity care but rather antenatal care only. Facilities studied included hospitals, maternity homes, health centers and clinics owned by government and private entities. Generally a hospital constitutes a high level facility with investigative laboratories, a range of health care workers (doctors, nurses, midwives and laboratory technicians among others), surgical theatres, blood bank and essential drugs and supplies. At this level of operation, hospitals are implicitly expected to be able to provide CEmOC. Maternity homes on the other hand may have most of what the hospitals have but not generally expected to have a surgical theatre or blood bank. Health centers and clinics are mainly outpatient facilities but occasionally operate like maternity homes by providing obstetric care. This categorization is very loose and bound to be misleading. For purposes of assessing quality of maternity services we used the eight signal functions as outlined in the guidelines for emergency obstetric care that has been proposed and used elsewhere [8].

Ethical clearance was obtained from the Kenya Medical Research Institute (KEMRI). Permission was also sought from the Ministry of Health and from the office of the Medical Officer of Health for City Council of Nairobi. Appointments to conduct the interviews were made with officers in charge of maternity units in the respective health facilities. Written informed consent was obtained for each of the respondents before the interview was conducted. The study was carried out between April and July 2006 by a clinical officer who underwent training for three days. A structured questionnaire was used for the interview to collect information on staffing, obstetric procedures performed, availability of equipment and supplies, referral system and health management information system.

## Results

Out of the 25 health facilities surveyed, 14 were located within the two slum areas and 11 were outside the slums but within Nairobi city. In total, there were four hospitals-including one government national teaching hospital, one government obstetric specialist hospital, one government district hospital, and one mission hospital. There were three health centers; one owned by government and the other two owned by non-governmental and community or faith based organizations. There were 13 maternity homes; one of them privately owned, two owned by non-governmental organizations, and 5 were private clinics. All four hospitals (a national referral and teaching hospital, an obstetric specialist hospital, a district hospital and a mission hospital) were located outside the slums.

### Availability of emergency obstetric services

Table 1 shows the emergency obstetric procedures performed by the 25 health facilities as well as the type of emergency obstetric care categories using the criteria noted earlier [8] Of the six basic emergency obstetric care procedures, assisted vaginal delivery was the least available with only 2 health facilities (both outside the slums) offering the service. Although 5 health facilities offered caesarean delivery, only two (national referral hospital and mission hospital) offered assisted vaginal delivery and thus met the criteria for comprehensive emergency obstetric care facility. Further, of the 5 health facilities that offered caesarean delivery, an additional two met the criteria for Comprehensive EmOC "minus 1" (excluding assisted vaginal delivery). Out of the remaining 21 health facilities, 10 were categorized as basic EmOC "minus 1" while 11 provided less than 5 basic emergency obstetric procedures. The dichotomy that categorizes facilities as either providing basic or comprehensive EmOC service has limitations as facilities in between are left out or their roles misrepresented. For example in spite of handling most obstetric emergencies and caesarean sections in this population, the specialist obstetric hospital was classified as offering less than basic EmOC since it does not offer assisted delivery, effectively putting it in the same category with the small private clinics.

**Table 1:** Procedures and type of emergency obstetric care in 25 health facilities, Korogocho, Viwandani and environs in Nairobi

Procedure/obstetric care category	Number	Percent
Procedure		
Parenteral antibiotics	24	96.0
Parenteral oxytocics	24	96.0
Parenteral anticonvulsants	21	84.0
Manual removal of retained placenta	18	72.0
Removal of retained products	17	68.0
Assisted vaginal delivery	2	8.0
Blood transfusion	11	44.0
Caesarean section	5	20.0
Type of EmOC		
Basic EmOC^a^	0	0.0
Comprehensive EmOC^b^	2	8.0
Basic EmOC "minus 1"^c^	10	40.0
Comprehensive EmOC "minus 1"^d^	2	16.0
Not applicable ^e^	11	44.0

^a ^A basic Emergency obstetric care (EmOC) facility the following six procedures:

parenteral antibiotics; parenteral oxytocics; parenteral anticonvulsants for

pre-eclampsia/eclampsia; manual removal of placenta; removal of retained products;

and assisted vaginal delivery.

^b ^A comprehensive EmOC facility provides all 6 basic EmOC procedures plus Caesarean

delivery and blood transfusion

^c ^Basic EmOC without assisted vaginal delivery

^d ^Comprehensive EmOC without assisted vaginal delivery

^e ^Does not meet the criteria for Basic EmOC "minus 1"

### Staffing

**Figure 1:**
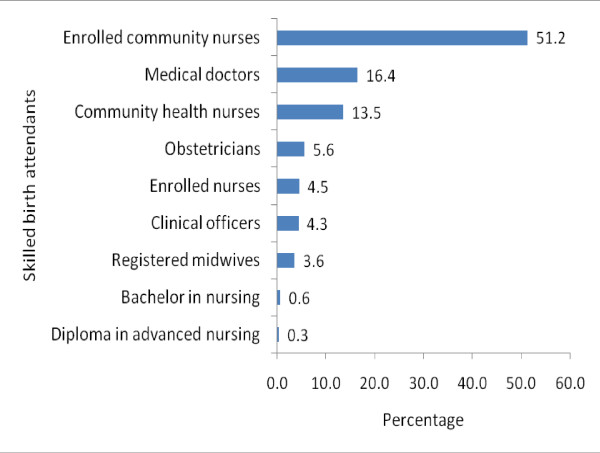
**Percent distribution of 646 skilled birth attendants in 24 health facilities, Korogocho, Viwandani and environs in Nairobi**.

There were a total of 646 skilled birth attendants in 24 health facilities (one health facility was run solely by a traditional birth attendant). Figure 1 presents the distribution of the types of skilled birth attendants. The term "skilled health worker" as defined by WHO refers to "an accredited health professional – such as a midwife, doctor or nurse – who has been educated and trained to proficiency in the skills needed to manage normal (uncomplicated) pregnancies, childbirth and the immediate postnatal period, and in the identification, management and referral of complications in women and newborns"[13]. Auxiliary nurses and traditional birth attendants whether trained or not have not been included in the category of skilled birth attendants. Slightly over half of all skilled attendants were enrolled nurses as opposed to 4% registered midwives. The latter are typically more qualified than enrolled nurses and the small proportion indicates a critical shortage of more qualified midwives. Out of the 36 obstetricians (6% of skilled birth attendants), 58% were in the national referral hospital and 17% in the obstetric specialist hospital. The way the data was collected could not allow assessment of skills per cadre. For example the respondent was asked "how many skilled birth attendants in this facility can carry out assisted vaginal delivery". Also majority of the skilled birth attendants fell in the broad category of midwife/nurse making breakdown by cadre not very useful. Majority of the skilled birth attendants could not perform some of the basic emergency obstetric procedures for example only 20% of the skilled health care workers could perform manual removal of retained placenta; 16% could do dilation and curettage; 9% could perform manual vacuum aspiration; and 8% could carry out assisted vaginal deliveries. These results demonstrate that even among skilled birth attendants, there is a skills shortage especially in the area of assisted delivery.

### Equipment and supplies

Adequate equipment and supplies are essential in the provision of quality emergency obstetric care. All health facilities surveyed use only disposable syringes and needles but some essential equipment and supplies were lacking in most facilities (Table 2). For instance, the manual vacuum aspirator is considered safer than curettes in the management of first trimester miscarriages but they were available in only 8 health facilities [14]. Further, some of the reported equipment were not in working condition or locked up somewhere. Of the 18 facilities that had speculum and curettes, less than half actually used them. In developing countries where cardiotocographs are not available for monitoring fetal heart rate and uterine contractions during labor, partographs (a simple graphical tool) are recommended to assess the progress of labor [15]. Only 12 health facilities had partographs available. Parenteral magnesium sulphate which is the drug of choice for eclampsia was available in only 7 health facilities [16,17].

**Table 2:** Percentage distribution of selected equipment and supplies in 25 health facilities, Korogocho, Viwandani and environs in Nairobi

Equipment/Supplies	Number of facilities N = 25	Percent (%)
*Equipment*		
Blood pressure gauge	22	88.0
Vacuum extractor	2	8.0
Manual vacuum aspirator	8	32.0
Curettes	18	72.0
Long arm gloves	6	24.0
		
*Supplies available*		
Parenteral antibiotics	22	88.0
Magnesium Sulphate	7	28.0
Anti-hypertensives	10	40.0
Parenteral anticonvulsants	17	68.0
Partograph, at last delivery	12	48.0

### Accessibility, referral, and quality assurance in maternity facilities

Although this study did not measure the physical distance to health facilities, slums are generally located within 10 km from the city center where most referral facilities are located. Even though the distance may not be long enough to be a barrier, accessing facilities at night can be difficult due to rampant insecurity. All the health facilities charge for obstetric services (Table 3). The fees for normal delivery ranged from 20 Kenya shillings in a government health center to about 5,500 (exchange rate was $1 = 72.5 KSh) in a mission hospital with an average of KSh 1,700 per delivery. The fee for caesarean delivery in the five health facilities that offer the service ranged from KShs 3,000 in a government district hospital to KShs 30,000 in a mission hospital. Considering the prevailing poverty in the slums, the cost of obstetric services especially in the private health facilities is unaffordable to most of the slum residents [18].

**Table 3:** Access to maternity care, referral and quality assurance in health facilities obstetric care: Maternal Health Facilities Survey, APHRC 2006, Nairobi

Variable	Number N = 25	Percentage (%)
*Accessibility factors*		
Facility opens less than 7 days a week	22	88.0
Personnel present 24 hours	20	80.0
Charge for normal delivery	25	100.0
Facility provides home deliveries	7	28.0
		
*Referral facilities*		
A printed referral form available	4	16.0
Telephone/radio call available	23	92.0
Ambulance available 24 hrs	5	20.0
		
*Quality control measures*		
Maternal death audit	13	52.0
Guidelines on infection control displayed	10	40.0
Different beds used at different stages of labor	19	76.0
Have a client register	19	76.0
HIV prophylaxis for personnel	6	24.0

A functioning referral and communication system allows timely transfer of obstetric emergencies. Almost all the health facilities had working telephones or shortwave radio (Table 3). Only 5 health facilities had emergency transport on site for referral of obstetric emergencies. The lack of ambulances was reflected in the high level of emergencies that arrive at referral hospitals (56%) on foot or public transport.

### Quality assurance

Although traditional birth attendants are not considered to be skilled birth attendants, they continue to operate on their own or with limited supervision. Three health facilities indicated that they had formal links with traditional birth attendants to offer them training and supervision. There was however no documentary evidence of these links. Many of the health facilities operate without proper supervision and regulation with 20% reporting no supervisory visit (from outside the health facility) in the last 12 month or more. Printed referral forms were found in only four health facilities (Table 3). Out of the 25 maternity facilities, only 13 (including all hospitals) conduct audit of maternal deaths and near misses (severe complications). Government facilities were more likely to conduct maternal audit than private owned facilities. It is standard practice that infection control guidelines should be displayed in the work place for quick reference, however only 40% of facilities had infection control guidelines displayed. It is expected that in countries with high HIV prevalence like Kenya [9], HIV post-exposure prophylaxis would be offered to health personnel who accidentally get exposed to HIV-infected blood (such as through a needle prick). Our results show that only 6 (24%) health facilities provide HIV post-exposure prophylaxis.

### Adequacy of obstetric records

Information generated from obstetric records can be useful in assessing use and quality of obstetric services. The obstetric records examined during the survey were largely incomplete. Five private clinics and maternity homes as well as the district hospital had no records on deliveries in 2005 (Table 3). Records on the number of deliveries, obstetric procedures performed, referrals, complications and maternal deaths in facilities with obstetric registers were not readily available at the time of the survey. Besides, over half of all complications and nearly half of maternal deaths in 2004–2005 did not have definitive diagnoses or causes of death, making usefulness of these data questionable.

The available records were nonetheless used to assess the case loads at the health facilities. Out of the total 41,112 deliveries in 2005, 86 percent occurred in three hospitals (the district hospital did not have data) indicating that the majority of normal deliveries, which should be taking place in lower level obstetric facilities did not. The obstetric specialist hospital was the busiest (with an average of 52 deliveries a day) in 2005 accounting for 46% of all deliveries recorded. Overall, the caesarean delivery rate of 20% appears quite high compared to the internationally recommended population-based rate of 5–15% (Table 4) [19]. Assisted delivery was just about 1.3% further confirming its low availability [8,20]. Additionally, the national referral hospital appeared to have a disproportionately higher case fatality (236 maternal deaths for 6,775 deliveries) compared to the obstetric specialist hospital (25 maternal deaths for 18,943 deliveries). It is important to note here that the national teaching hospital handles most of the complicated cases referred from other facilities in the country and this might explain the high case fatality rate in this particular hospital. A study by Magadi et al. on maternal mortality in Kenyan hospitals observed that Pumwani hospital, the specialist obstetric hospital in Nairobi was better equipped than most hospitals and recorded lower maternal mortality than other hospitals [19]. These explanations notwithstanding, there might be need to examine further why there are huge differences observed in the two hospitals in Nairobi city. There is a also a small possibility that the observed results are a function of poor/incomplete records resulting into wrong estimates for either facility.

**Table 4:** Caesarean delivery rates in 4 health facilities with records of caesarean deliveries in 2005, Korogocho, Viwandani and environs in Nairobi^1^

Health facility	Deliveries	Cesearean deliveries	Caesearean delivery rate (%)
Nursing home	241	1	0.4
Obstetric specialist hospital	18,943	2,802	14.8
Mission hospital	9,717	3,289	33.8
National referral and teaching hospital	6,775	2,149	31.7
Total	35,676	8,241	23.1

^1^The caesarean rate for all 25 facilities is 20.0% (8,241/41,112)

## Discussion

Reducing maternal mortality remains a big challenge facing the attainment of MDG5. It is generally agreed that maternal health care has not improved significantly since the MDGs were set. Access to quality and timely emergency obstetric care is crucial as most obstetric complications are unpredictable and yet life threatening. Although data from this study is from a small geographical area, it sheds light to the often forgotten sub-population of the urban poor who like the rural folks encounter barriers in accessing obstetric care. From the foregoing results, it is apparent that emergency obstetric services offered at the health facilities assessed were not optimal. Many facilities lacked essential equipment and many health providers did not have critical skills needed to conduct deliveries with minor complications and yet all professionally deployed staff are thought to be skilled enough to manage normal deliveries and obstetric complications. The figures reported in surveys for example, the proportion of women who delivered with assistance of skilled personnel might be higher than the real capacity available to provide safe obstetric care. In service training might go a long way to ensuring that staff have the necessary basic skills to perform their duties.

In this study assisted vaginal delivery was found to be a rare procedure. The KSPA also reported that only 9% of maternity health facilities in Nairobi province offered assisted vaginal delivery (7% nationally) while the proportion for caesarean delivery was 24% in Nairobi and 9% nationally [9] compared to 20% found in this study. The finding of low assisted vaginal delivery rate of 1.3% in this study indicates that perhaps unnecessary caesarean sections are carried out for cases such as prolonged second stage of labor where assisted vaginal delivery would have been indicated [20-22]. This should be looked at in the light of considerably higher cost of caesarean sections and the risks associated with surgical procedures. It is also important to consider other issues such provider preference and how this affects the characterization of service delivery levels and its impact on overall reporting on maternity health care performance. The absence of assisted vaginal delivery is why we categorized facilities as "basic or comprehensive EmOC facilities minus 1", in addition to the standard convention. We speculate that provider bias against assisted delivery might also partly explain this scenario but indeed further research is needed to assess the position of assisted delivery in obstetric care in Kenya.

Whereas good records can be a vital source of information for monitoring the provision of obstetric care, the largely incomplete obstetric records in this study posed a major challenge in drawing conclusions from the data. Nevertheless, from the available records for 2005, some important observations were made. For example the obstetric specialist hospital (which did not meet criteria for basic EmOC) had more deliveries and yet lower maternal deaths compared to the national referral hospital (classified as comprehensive EmOC). This anomaly might be the result of inaccurate records but could also indicate that using the Maine et al [8] criteria does not necessarily differentiate low and high quality emergency obstetric care facilities. It could also as well be the case that the national hospital handles most of the late complications from other lower facilities, hence a higher risk of death. This underscores the need for standardization of records in terms of completeness and accuracy of information to allow evidence-based decision making at sub-national and nationals levels.

Accessibility to obstetric care remains a challenge. Although the distance between the slums and many of the fairer health facilities is not a big barrier as compared to rural settings, insecurity in the slums especially at night makes accessibility very difficult and people resort to using facilities near to them irrespective of their quality. Obstetric services in Kenya are paid for. Most people especially the poor who are not in formal employment pay for medical care out of pocket. Others who are in formal employment have access to medical insurance through their employers although many insurers don't cover obstetric risk. The big difference in cost for both normal deliveries and caesarean sections between government and private facilities is because government provides obstetric services at a subsidized cost. This incentive results into overcrowding in government facilities, and hence compromised quality. The shortfall in service might partly be the reason why private providers including TBAs continue to thrive in this community.

Given the centrality of emergency obstetric care services in reducing maternal morbidity and mortality, for reasonable improvements to be made there is need for increased availability, use, and quality of emergency obstetric care services for the growing slum population. In addition to increasing availability of obstetric facilities, it is imperative to regulate and supervise private and public providers to provide a minimum package of quality obstetric services. This might encourage women to seek delivery services at the lower level facilities so that the higher level facilities focus more on complicated cases.

In several ways this study could not answer some of the pertinent issues related to obstetric care and these are outlined below as limitations to this study. Health care seeking choices and patterns in an urban area especially in an informal settlement is very difficult to map out. Some individuals choose to deliver in their rural homes or go to facilities they consider affordable not necessarily those nearest to them. As such estimating a catchment population is very difficult unless all facilities in the city are studied. We were not able to compute indices that require the catchment population such as proportion of all births in emergency obstetric facilities, met need for obstetric complications, caesarean deliveries as a proportion of all births and case fatality rates for community wide deliveries. These indicators would have added valuable information for service improvement.

## Conclusion

The quality of emergency obstetric care services in Nairobi slums is poor. Essential equipment, supplies, skilled trained personnel, and other support services are in short supply especially in maternity facilities located within the slums. There is also little supervision to ensure adherence to standards especially among the small private maternity facilities. Medical records even in big health facilities are poorly kept and very difficult to use. Computerization of medical records will go a long way in making medical records more usable.

## Competing interests

The authors declare that they have no competing interests.

## Authors' contributions

AKZ took lead in preparing the manuscript. He participated in designing the study, data collection supervision, data cleaning, and conducted most of the analysis and writing. SM contributed to study design, data analysis, manuscript preparation, and interpretation of findings. NM conceptualized the study; she supervised data collection and analysis and wrote sections of this paper. TS contributed to the design and data collection supervision. JCF contributed to the conceptualization of the study, manuscript preparation, and interpretation of findings.

## Pre-publication history

The pre-publication history for this paper can be accessed here:


